# 

**Published:** 1882-04

**Authors:** 


ESTABLISHED IN 1855.

OLD SERIES. Vol. XX—No. 1. | APRIL, 1882. | NEW SERIES Vol. 1—No. 7

### ATLANTA
### MEDICAL REGISTER.

*(ATLANTA MEDICAL AND SURGICAL JOURNAL.)*

EDITORS:

JNO. THAD. JOHNSON, M.D., JAMES B. BAIRD, M.D.

PUBLISHED BY H. H. DICKSON, AT $2.50 A YEAR IN ADVANCE

ATLANTA, GEORGIA:

*H. H. DICKSON, BOOK AND JOB PRINTER AND BOOKBINDER.*

1882.

